# Parasitism by the Endoparasitoid, *Cotesia flavipes* Induces Cellular Immunosuppression and Enhances Susceptibility of the Sugar Cane Borer, *Diatraea saccharalis* to *Bacillus thuringiensis*

**DOI:** 10.1673/031.011.11901

**Published:** 2011-09-17

**Authors:** A.M.A. Mahmoud, E.J. De Luna-Santillana, M.A. Rodríguez-Perez

**Affiliations:** Centro de Biotecnologia Genomica (CBG), Instituto Politécnico Nacional (IPN), Mexico and Centro de Investigación en Ciencia Aplicada y Tecnología Avanzada (CICATA) (Unidad Altamira), Mexico

**Keywords:** cellular immunity, encapsulation, nodulation, polydnavirus

## Abstract

*Cotesia flavipes* Cameron (Hymenoptera: Braconidae), is a gregarious larval endoparasitoid of the sugarcane borer, *Diatraea saccharalis* Fabricius (Lepidoptera: Crambidae). The aim of this research was to analyze cellular immunosuppression of *D. saccharalis* parasitized by *C.* *flavipes* in terms of encapsulation, melanization, and hemocyte nodule formation. The encapsulation assay was done 1 and 6 days after parasitoid oviposition. In addition, the susceptibility of parasitized and nonparasitzed larvae to *Bacillus thuringiensis* HD 73 strain was assessed. 3, 12, and 24 h after bead injection; the percentages of encapsulation were significantly higher in unparasitized larvae compared to larvae parasitized 1 and 6 days after oviposition. Interestingly, there was a significant reduction in numbers of beads encapsulated at 1 day after oviposition compared to 6 days, and unparasitized larvae. The percentage of melanized beads decreased significantly in parasitized larvae compared to control. There was a reduction in the number of nodules in parasitized larvae compared to unparasitized controls. Larvae that were injected with polyndavirus 24 h before beads were injected showed significantly reduced encapsulation responses relative to control larvae. The *D. saccharalis* parasitized by *C.* *flavipes* exhibited higher susceptibility to *B. thuringiensis.* These results suggest that parasitization induced host immunosuppression, and the immunosuppression factors could impair the defense capacity against microbial pathogens - causing an increase in pathogen susceptibility.

## Introduction

The sugarcane borer, *Diatraea saccharalis* (Fabricius) (Lepidoptera: Crambidae), is a key pest of sugarcane in the Americas (Long and Hensley 1972; Posey et al. 2006; Reagan 2001). Occasionally, this insect also causes economic losses in rice and grain sorghum (Castro et al. 2004). *Cotesia favipes* (Cameron) (Hymenoptera: Brachonidae) is a koinobiont, gregarious, larval endoparasitoid used in biocontrol programs of *D. saccharalis* (Botelho and Macedo 2002). *Cotesia flavipes* parasitizes *D. saccharalis*, and induces host immunosuppression. Insects utilize both humoral and cellular defense responses against invading pathogens and parasites. Humoral defense responses include the production of antimicrobial peptides (Lowenberger 2001), reactive intermediates of oxygen or nitrogen (Bogdan et al. 2000), and the prophenoloxidase activating system that regulates coagulation or melanization of hemolymph (Gillespie et al. 1997; Kanost et al. 2004). Cellular defense responses refer to hemocyte-mediated immune responses including phagocytosis, nodulation, and encapsulation (Lavine and Strand 2002). Small pathogens such as bacteria and fungi are generally killed by humoral defense responses or phagocytosed by hemocytes, whereas parasites such as parasitoids and nematodes are encapsulated by hemocytes (Schmidt et al. 2001). Successful parasitization by endoparasitic wasps requires suppresion of the immune system of the host to prevent encapsulation of the wasp's egg, and developmental arrest of the host to divert host nutrients to support parasite development (Beckage and Kanost 1993; Strand and Pech 1995a). Some hymenopteran wasps possess both maternal and embryonic immunosuppressive factors (Theopold et al. 2000). It has been shown that maternal factors include ovarian proteins, venom, and polydnavirus (Amaya *et al.* 2005; Asgari et al. 1997; Bae and Kim 2004; Beckage 1998; Glatz et al. 2004; Morales et al. 2005; Richards and Parkinson 2000); while embryonic factors include teratocytes, a specific cell type derived from the embryonic serosal membrane (Basio and Kim 2005; Dahlman and Vinson 1993; Jones and Coudron 1993; Krell et al. 1982; Webb and Luckhart 1994).

Polydnaviruses have been found in two families, the Braconidae and Ichneumonidae, and are classified into bracovirus and ichnovirus, respectively (Webb et al. 2000). Polydnaviruses are unique viruses having circular, double-stranded DNA and are segmented (Web and Strand 2005; Webb et al. 2000). Polydnaviruses persist as stably integrated proviruses in the genome of certain parasitoid species and replicate in the calyx cells in the ovaries where virions accumulate to high concentrations (Wyler and Lanzrein 2003). The hosts of polydnavirus-carrying wasp species are primarily larval stage Lepidoptera. When a female wasp oviposits into a host, she injects one or more eggs and the virions that infect host immune cells of the host tissues (Schmidt *et* al. 2001; Stoltz 1993). Polydnaviruses don't replicate in the host, but expression of viral genes prevents the host immune system from killing the wasp's egg and causes other physiological alterations that ultimately cause the host to die (Asgari et al. 1996; Beckage and Gelman 2004; Turnbull and Webb 2002; Webb and Strand 2005). Thus, a mutualism exists between polydnaviruses and certain wasp species as viral transmission depends on parasitoid survival, and parasitoid survival depends on viral infection of the host (Webb et al. 2006).

The aim of this research was to analyze cellular immunosuppression of *D. saccharalis* parasitized by *C. flavipes* in terms of encapsulation, melanization, and nodulation responses. We also hypothesized that the immunosuppressive factors of *C.* *flavipes* could enhance the susceptibility of *D. saccharalis* larvae to *Bacillus thuringenisis.*

## Materials and Methods

### Insect rearing

*Diatraea saccharalis* larvae were reared on an artificial medium and staged to instar by examining the width of the head capsule prior to exposing them to parasitoids. The artificial diet (BioServ product No. f9775B, www.bioserv.com) was made following the manufacturer's instructions. In brief, the diet was mixed at high speed with melted sterile bacteriological agar-agar (200g/liter) using a commercial blender. The diet was poured rapidly into plastic cups or Petri dishes and left at room temperature to cool and solidify for at least one hour. The diet was stored at 4° C until needed. Prior to adding larvae to the dishes, the diet surface was scored with a needle.

*D. saccharalis* and *C. flavipes* were reared at 28° C under 16:8 L:D photoperiod conditions in an incubator. To generate the adult moth stocks, *D. saccharalis* pupae were collected daily and placed inside glass or plastic containers lined with a plastic bag as a substrate for egg laying. Small cups filled with 10% sucrose solution secured with a cotton plug were provided for the emerged adult moths, which lived approximately 5–7 days. The adult moths laid light yellow egg clusters on the plastic bag. The eggs clusters were collected and left to mature in the Petri dish, where the color of the eggs turned from yellow to orange to black over a period of 2–5 days. Once the eggs clusters darkened, they were transferred to a fresh diet plate. The eggs hatched within 1–2 days and the larvae migrated into the scored tracks made on the surface of the diet. The larvae were transferred to fresh diet plates as needed to prevent crowding as the larvae grew until they pupated (25–30 days). The pupae were then collected from the dishes and placed into the adult-rearing cages.

Third to sixth instars of *D. saccharalis* larvae were used to generate the stocks of *C. flavipes* colony. For parasitization, each larva was exposed individually to a single; 1–3 day-old mated female parasitoid, to ensure that successful parasitization occurred. After a single sting the larva was transferred to new Petri dishes containing new diet. Larvae were transferred to fresh diet every 4–5 days until *C.* *flavipes* emerged and spun cocoons adjacent to the host larva. Parasitoid larvae usually emerged from five or six instar *D. saccharalis* larvae and the host does not survive. Parasitoid *C. flavipes* cocoons were collected and transferred to a centrifuge tube containing a piece of moist cotton at the bottom, and a drop of honey in the inner wall for feeding the adult parasitoids. The tubes were kept in an incubator at the same environmental conditions mentioned above. After emergence, about five males and five females' parasitoids were allowed to mate for 24 h and the females were then used for parasitization of larvae. After 3–4 days, the adult parasitoids were transferred to a new centrifuge tube with new food. All solutions, glassware, and plastic materials used in this study were sterilized.

### Encapsulation assay

**Figure 1.**
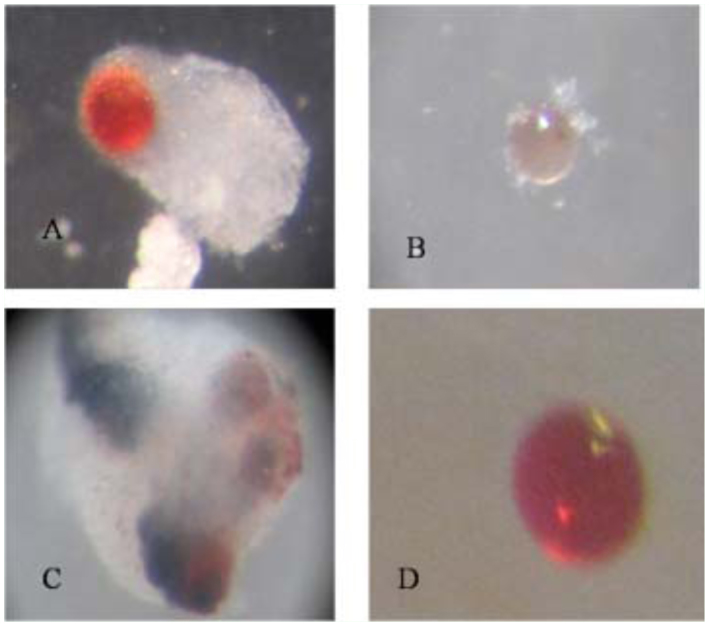
Photomicrographs of Sephadex A-25 beads after recovery from *Diatraea saccharalis* larvae showing the scoring system for encapsulation: (A) encapsulated bead; (B) bead with different adherent cells; (C) melanized beads; (D) bead with no adherent cells. High quality figures are available online.

**Figure 2.**
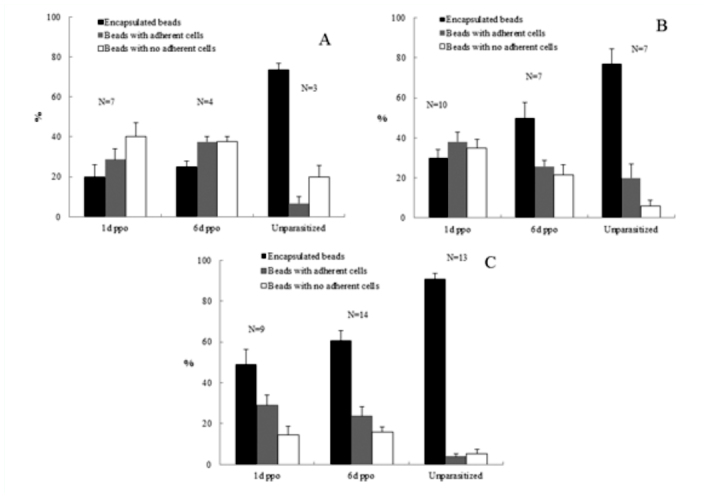
Effects of parasitization on encapsulation responses of *Diatraea saccharalis* larvae to Sephadex A-25 beads. Each larva received 10–15 beads, and 10 beads were recovered and scored for encapsulation. Graph bars represent mean percentages (of n larvae) ± standard error. ppo = post-parasitoid oviposition (A). Beads were recovered (by dissection) from larvae after 3 h. (B) Beads were recovered after 12 h. (C) Beads were recovered after 24 h. High quality figures are available online.

**Figure 3.**
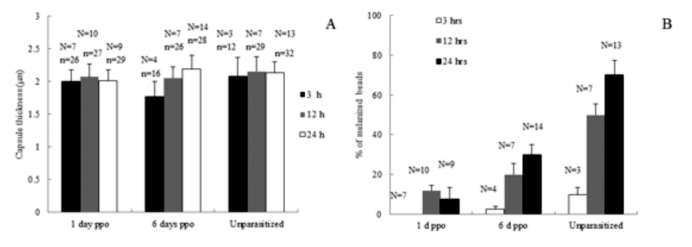
Effects of parasitization on capsule thickness (A) and melanization (B) of Sephadex A-25 beads recovered after incubation (3, 12, or 24 h) in the hemocoel of fifth-instar *Diatraea saccharalis* larvae. (A) Graph bars represent mean thickness ± standard error of capsules surrounding n beads from n larvae. (B) Graph bars represent mean percentage (± standard error) of beads (from n larvae) showing associated melanization. High quality figures are available online.

Sephadex A-25 beads, sterilized in 95% alcohol, were used to stimulate encapsulation. Larvae were divided into three treatment groups at the time of bead injection: larvae parasitized 24 h earlier (1 day after oviposition), larvae parasitized 6 days earlier (6 days after oviposition), and nonparasitized larvae (control). Larvae were parasitized by exposure to female *C. flavipes* as new fifth instars for both 1 and 6 days after oviposition until one oviposition was observed. The A-25 beads were stained in a 0.1% congo red solution to aid in recovery after injection (Lavine and Beckage 1996). Beads were dried under UV light in a sterile tissue culture hood, and re-suspended in phosphate buffer saline (PBS) (100 mM pH 7.0). Larvae were anesthetized on ice, and beads were injected through the dorsal line into the hemocoel using a Hamilton 7000 series microsyringe. Ten to fifteen beads were injected/larva, in a total volume of 5µl PBS/injection. Groups of larvae were dissected 3, 12, and 24 hrs later to assess encapsulation and verify the presence of parasitoids in the hemocoels of larvae that had been parasitized 6 days before bead injection. Beads were examined under a stereomicroscope at 50-fold magnification and assigned to one of the following categories based on the attendant hemocytic encapsulation response: (1) encapsulated beads with a clear, thick capsule (Figure 1A); (2) beads with adherent cells, but without a clear capsule (Figure 1B); (3) melanized beads (Figure 1C); or (4) unencapsulated beads with no obvious adherent cells (Figure 1D). ‘Encapsulated’ was collectively defined as varying from a complete covering by a thin cellular layer (weak encapsulation) to a 50–100% covering by a multicellular layer (strong encapsulation). It was presumed that one measure of the avidity of the hemocyte response might be reflected in capsule thickness, which was measured using a (Dinolite Digital Microscope AN-413T, www.dinolite.com). If a bead showed any visible evidence of melanin deposition on its surface or within the capsule layers, it was scored as melanized.

### Polydnavirus purification and injection

Polydnavirus was filter-purified from dissected adult *C. flavipes* ovaries according to the method used by (Beckage et al. 1994). Briefly, 2 or 3-day-old female adults were used to obtain calyx fluid from the ovary. *C. flavipes* were individually swabbed with 95% ethanol, and then transferred into PBS (100 mM, pH 7.0) in a 1.5 ml microcentrifuge tube on ice. Ovaries of female were removed with the aid of a dissecting microscope and placed in a microcentrifuge tube containing PBS on ice and were homogenized by drawing the suspension through a 23-gauge needle approximately eight times. Venom glands were carefully excluded from the preparation during dissection. Following homogenization, the polydnavirus suspension was centrifuged 3 min at 1500 *g* at 4° C through a 0.45 µm filter (Phenomenex, www.phenomenex.com) to remove tissue fragments. The ovary filtrate was then centrifuged for 15 min at 15,000 *g* at 4° C. The pellet was resuspended in PBS. For bioassay experiments, 70% ethanol surface-sterilized nonparasitized fifth instar *D. saccharalis* larvae were injected with 5 µl PBS (=1 wasp ovary equivalent) of *C.* *flavipes* polydnavirus, or PBS as a control via the dorsal line using a sterile 30 gauge Hamilton syringe. Twenty-four hours after injection of polydnavirus, each larva was injected with 10 –15 Sephadex A-25 beads, which were dissected and scored for encapsulation as mentioned above.

### Nodulation assay

To determine hemocyte nodule formation, late-parasitized or nonparasitized fifth instar *D. saccharalis* larvae were surface-sterilized with 70% ethanol and chilled on ice. Parasitized or nonparasitized larvae were injected with 5 µl heat-killed *Eschericia coli* (1 × 10^6^ cells/larva) using a sterile 30 gauge Hamilton syringe, and incubated at 28° C in an incubator. The number of nodules was counted at 24 h post injection. The larvae were dissected out by opening the hemoceol. Melanized and dark nodules on gut, fat body, and Malpighian tubules were counted under a stereomicroscope at 50-fold magnification. Also, the darkened, melanized nodules present on the ventral side of the abdomen and thoraxes were also counted externally through the transparent cuticle before dissection.

### Susceptibility assay

Preliminary experiments of different *B. thuringenisis* strains (HD 133, HD 551, and HD 73) against *D. saccharalis* neonate larvae indicated that the HD73 strain is the most toxic strain. Fifth instar larvae of *D. saccharalis* were divided into two groups, parasitized with *C. flavipes* and unparasitized, and starved for 12 h before use in the experiment. The parasitized and nonparasitized larvae were fed on contaminated diet containing (500 µg Bt/ml diet). Each larva was kept individually in a plastic cup, and the percentage of larval mortality was calculated. This experiment was replicated three times with 10 larvae per replication.

### Statistical analyses

All data were analyzed using the SPSS11.0.0 software (SPSS Inc. 2001). Data of encapsulation, melanization, and capsule thickness were analyzed using one-way ANOVA; post-hoc differences between treatment pairs were examined using Duncan Multiple Range test (DMR-test). However, nodulation and susceptibility assays were analyzed by a least Squared difference (LSD) test and discriminated at Type error = 0.05. Means different at the 0.05% level were considered significantly different.

## Results

### Effects of *C. flavipes* parasitism on host cellular immune capacity

**Figure 4.**
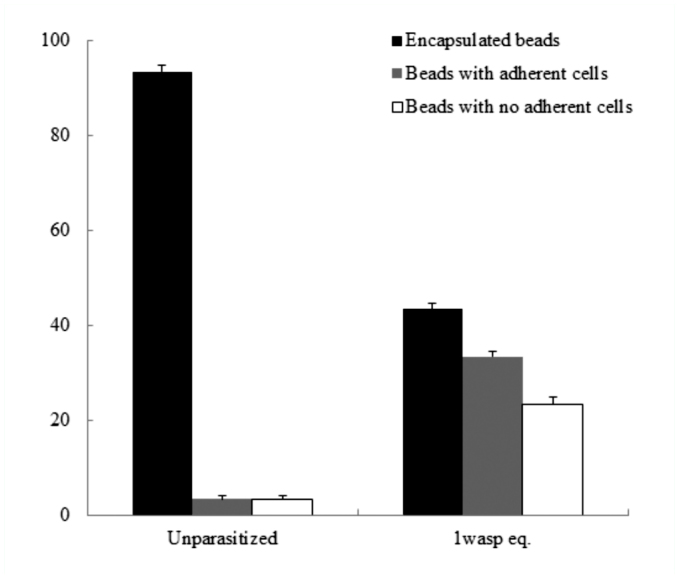
Effects of filter-purified polydnavirus on encapsulation responses of fifth-instar *Diatraea saccharalis* larvae to Sephadex A-25 beads. Larvae received I wasp equivalent (eq.) of filtered calyx fluid. Twenty-four hours later, each larva received 10–15 beads, which were recovered after a 24 h incubation. Graph bars represent mean percentages (of n larvae) ± standard error. High quality figures are available online.

In this study, cellular immune capacity of the parasitized host larvae was analyzed by encapsulation and hemocyte nodule formation. Hemocyte encapsulation was analyzed *in vivo* in parasitized and nonparasitized *D. saccharalis.* Encapsulation by the hemocytes was determined based on the observation of white hemocyte capsules formed around the injected beads and also presence of melanized beads (Figure 1). With increasing time, the percentage of encapsulated beads increased and the extent of encapsulation as well as the percentage of melanized capsules also increased gradually (Figures 2 and 3). The encapsulation response of the 1 and 6 days after oviposition larvae to Sephadex A-25 beads always was significantly reduced (*p* < 0.05) relative to unparasitized larvae. This was true regardless of whether the beads remained in the caterpillar host for 3, 12, or 24 h (Figure 2A, B, C), respectively. No significant difference in encapsulation was found between larvae either parasitized 1 or 6 days after oviposition at 3 h post injection, however, the percentage of encapsulated beads at 12 and 24 h post injection increased significantly in 6 days after oviposition compared to 1 day after oviposition (Figure 2A, B, C). The percentages of beads showing few adherent hemocyte cells at 3 and 24 h post injection increased significantly in 1 and 6 days after oviposition compared to unparasitized larvae; however, no significant different was found at 12 h post injection in all the treatments (Figures 2A, B, C). The thicknesses of the capsules were variable and some of them were thicker than the diameter of the beads (Figure 1). Interestingly, capsule thickness did not vary significantly with *in vivo* incubation period (*p* > 0.05), nor across treatment groups (*p* > 0.05) (Figure 3A). There were few beads associated with melanization at 3 h post injections in both parasitized and control larvae (*p* > 0.05) (Figure 3B). However, the percentage of beads showing melanization at 12 and 24 h post injections was significantly reduced (*p* < 0.05) in the 1 day after oviposition and 6 days after oviposition larvae compared to the unparasitized larvae (Figure 3B). Also the percentage of melanized beads increased significantly in the 6 days after oviposition compared to 1 day after oviposition at 24 h post injection. Larvae that were injected with filter-purified polydnavirus 24 h before beads were injected showed significantly reduced encapsulation responses relative to control larvae given PBS only (*p* < 0.05) (Figure 4).

**Figure 5.**
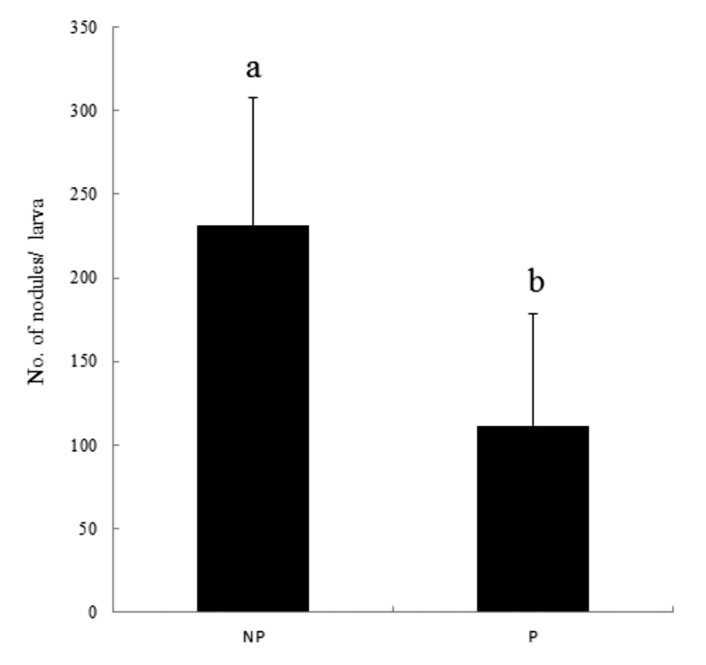
Effect of *Cotesia flavipes* parasitization on hemocyte nodule formation of *Diatraea saccharalis* in response to nonpathogenic bacteria, *E. coli*, infection (5×10^6^ cells/larva). Each measurement was replicated 10 times. ‘NP’ and ‘P’ represent nonparasitized and parasitized larvae, respectively. Error bars represent standard deviations. Different letters above error bars indicate significant difference at a = 0.05 (LSD test). High quality figures are available online.

The larvae parasitized by *C. flavipes* had significantly lower nodule formation than the nonparasitized larvae (Figure 5). In *D. saccharalis*, the nonparasitized larvae could form an average 231.44 nodules in response to bacterial infection, while the parasitized larvae averaged 111.5 nodules (Figure 5).

### Susceptibility assay

**Figure 6.**
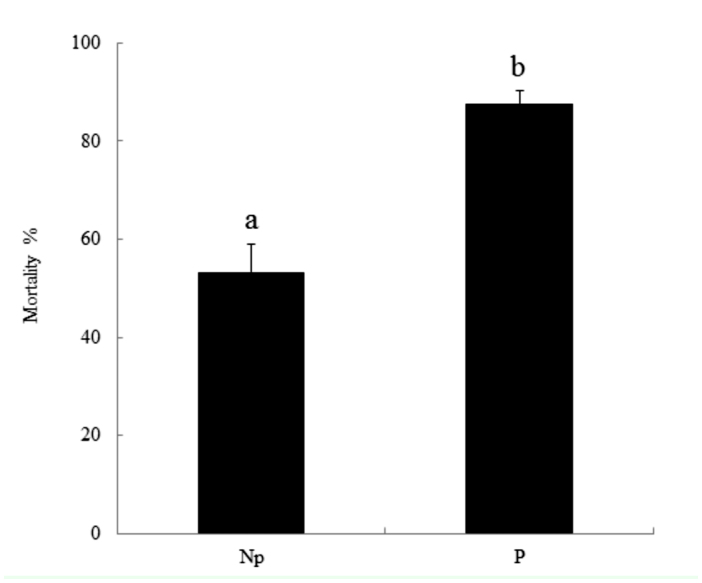
Parasitism by *Cotesia flavipes* enhances pathogen susceptibility of *Diatraea saccharalis.* Synergistic pathogenicity between *Bacillus thuringiensis* HD73 strain (500 Mg/ml diet) and the parasitism. Each concentration was replicated three times with 10 larvae per replication. Error bars represent standard deviations. Different letters above indicate significant different among means at Type I error = 0.05 (LSD test). High quality figures are available online.

Parasitized *D. saccharalis* showed significantly higher susceptibilities to the oral *B. thuringenisis* HD 73 strain infection than the nonparasitized (Figure 6). The mean percentage of larval mortality was 88% and 53% for unparasitized and parasitized larvae, respectively (Figure 6). Controls that were not treated with Bt did not show any mortality in either parasitized or nonparasitized *D. saccharalis.*

## Discussion

Parasitization of lepidopteran larvae by endoparasitoids is usually associated with suppression of host cellular and humoral immunity (Glatz et al. 2004). Parasitization by *Cotesia* spp. induces host immunosuppression in several insect species (Schmidt 2007; Amaya et al. 2005; Schmidt et al. 2005; Bae and Kim 2004; Hayakawa 1994; Stoltz 1993). This study evaluated the effects of parasitization by *C. flavipes* on cellular immune response of *D. saccharalis* in terms of two cellular immune reactions: encapsulation and hemocyte nodule formation. Encapsulation is the main cellular reaction of host insects against the endoparasitoids, as illustrated by nonpermissive host insects in which hemocytes attach and spread across the developing parasitoid to form a multilayered sheath of cells (Strand and Pech 1995b). In hemocyte encapsulation, both granular cells and plasmatocytes are required for capsule formation (Pech and Strand 1996). Granular cells are the first hemocytes to attach to a foreign target, which is followed by the attachment of plasmatocytes to the primary granular cell coat. Finally granular cells form the outermost layer, at which point the capsule ceases to grow in size (Cai et al. 2004; Pech and Strand 1996).

In our results there were strong reductions in percentages of encapsulated beads and in the number of nodules formed in parasitized larvae compared to unparasitized. Interestingly, hosts exhibit a recovery of their hemocytic encapsulation capabilities at 12 and 24 h post bead injection in 6 days after oviposition compared to 1 day after oviposition. The mechanisms responsible for suppression of encapsulation have been studied in many parasitoid/host systems (Hu et al., 2003; Ibrahim and Kim, 2006; Lavine and Beckage 1996; Pech and Strand 1996). Polydnaviruss have been shown to mediate several alterations of host physiology (Beckage 1993), one of which is immunosuppression (Lavine and Beckage 1995; Strand and Pech 1995a, b), that not only protects the parasitoid egg(s) from encapsulation, but also prevents a host response to other biotic and abiotic targets which would normally be encapsulated or nodulated (Davies et al. 1987; Davies and Vinson 1988; Guzo and Stoltz 1987; Stoltz and Guzo 1986; Strand and Noda 1991; Vinson 1974; Tanaka 1987). In *Cotesia kariyai*, the laid eggs can be protected from the encapsulation reaction by an ovarian immuno-evasive protein encoded in the wasp genome (Tanaka et al. 2002). Venom, as another host immune-suppressive factor, acts alone in *Cotesia melanoscela* (Stoltz et al. 1988) or synergistically in *Cotesia glomeratus* (Kitano 1986), though its effect may be dispensable in *Campoletis sonorensis* presumably because of the shared gene structures with polydnavirus (Webb and Summers 1990). Moreover, several polydnaviral genes are suggested to be implicated in suppression of encapsulation (Edson et al. 1980).

Ibrahim and Kim, (2006) classified these candidate genes into two groups in terms of their inhibitory strategies to manipulate adhesiveness of the hemocytes in the processes of encapsulation. One is to interrupt a normal cytoskeletal rearrangement in response to pathogen infection, as suggested by the VHV1.1 gene of *C.* *sonorensis* ichnovirus (Li and Webb 1994) and the CrV1 gene of *Cotesia rubecula* bracovirus (Asgari et al. 1996). The other is to hinder the functional interaction between the hemocyte receptor and foreign ligands by depressing the inducible expression of selected a- and b-integrin, or by forming a physical barrier such as the GIc1.8 of *Microplitis demolitor* bracovirus (Beck and Strand 2005; Lavine and Strand 2003). In our experiment it was found that the suppression of the encapsulation response to Sephadex beads was induced by injection of polydnavirus into nonparasitized *D. saccharalis* larvae, suggesting the effect is virally mediated. Lavine and Beckage (1996) found that the immunosuppressive and morphological hemocyte changes were mimicked by injection of unparasitized *Manduca sexta* larvae with filter-purified *C. congregata* polydnavirus, indicative of viral mediation of these changes. Temporal expression of some polydnavirus genes (Harwood and Beckage 1994; Harwood et al. 1994) correlates with the observed pattern of hemocyte morphological abnormalities and immunosuppression, further suggesting that the immunosuppression is a result of transcription/translation of polydnavirus genes (Lavine and Beckage 1996).

In the presence of the preconditioning effects of the polydnavirus, as well as other factors normally present in naturally parasitized hosts (such as ovarian proteins, venom, and teratocytes) one day after oviposition larvae clearly exhibited a reduced encapsulation response to A-25 beads relative to the 6 days after oviposition. One day after oviposition larvae did show low numbers of hemocytes with normal morphologies (unpublished data), and such larvae are sometimes able to encapsulate small numbers of beads; when this occurred such capsules were the same thickness as capsules found in 6 day after oviposition or unparasitized larvae. Thus, the newly parasitized larvae probably retain a small population of functioning hemocytes.

Our results indicate that there was a near total recovery of the host's response to Sephadex A-25 beads occured by 6 days after oviposition, even though the developing parasitoids remain unencapsulated. Lavine and Beckage (1996) indicated that 24 h after parasitization by the braconid parasitoid *C. congregata, M. sexta* larvae showed a strong suppression of their encapsulation response to Sephadex A-25 beads and by 8 days after oviposition the frequency of encapsulation of injected beads was no longer distinguishable from that observed in nonparasitized controls. Ross and Dunn (1989) also observed a recovery of host *M. sexta's* ability to clear P*seudomonas aeruginosa* and *E. coli* by 10 days after oviposition. However, recovery never reached control levels, and *P. aeruginosa*-*induced* host mortality remained high. Thus, the host immune response may remain partially impaired despite recovery of some capabilities, i.e., encapsulation of beads. The significant increase in the numbers of beads showing few adherent cells in parasitized larvae was reported by Lavine and Beckage (1996). They found that parasitism of *C.* *congregata* provoked radical morphological alterations in *M. sexta* hemocytes that were correlated with their reduced competence to encapsulate beads. These alterations included clumping, loss of adherence, and blebbing, which are probably manifestations of the inability of host hemocytes to respond to objects that would normally be encapsulated.

The reduced level of hemolymph defensive melanization in the parasitized host has been shown to be accompanied by reduced activity of phenoloxidase (Lavine and Beckage 1995). The significantly reduced percentage of beads showing melanization in the 1 day after oviposition larvae probably was a consequence of the very low numbers of beads encapsulated in these larvae, since melanization did not occur in the absence of encapsulation, suggesting that melanization of foreign bodies requires hemocytes for induction. It has been mentioned that polydnavirus of *C.* *sonorensis* plays a significant role in blocking host plasma melanization and hemocyte-spreading behavior (Luckhart and Webb 1996; Shelby et al. 2000).

These results suggest that the *C. flavipes* parasitization induces immunosuppression in *D. saccharalis*. The immunosuppressive factors of the endoparasitoids include polydnavirus, venom, ovarian proteins, and teratocytes. Immunosuppression could impair insect defense capacity against microbial pathogens, which results in an increase of pathogen susceptibility. Using this hypothesis, our research has tested whether one pathogen, *B. thuringenisis* can increase its pathogenicity in the immunodepressed hosts. It was found that the *D. saccharalis* larvae parasitized by *C.* *flavipes* exhibited higher mortality to *B. thuringenisis* HD 73 strain than in unparasitized larvae. These results are similar to results reported by Jung et al. (2006) who found that *Plutella xylostella* parasitized by either *C. plutellae* or *C. glomerata* exhibited higher susceptibility to an entomopathogenic bacterium, *Xenorhabdus nematophila* (Xn), and a viral pathogen, *Autographa californica* nucleopolyhedrosis virus (AcNPV), than the nonparasitized. It has been reported that polydnavirus is the main immunodepressive factor that is responsible for inducing the host immunosuppression (Dushay and Beckage 1993). Three *C. plutellae* polydnaviral genes were tested by Jung et al. (2006) and indicated that CpBV-Lectin did not significantly increase the pathogen susceptibility. However, two CpBV15 genes significantly increased the pathogen susceptibility, in which CpBV15β construct was more potent than the CpBV15α. These results suggest that the polydnaviral genes are associated with immunodepression, that results in the significant induction of pathogen susceptibility of *P. xylostella.* Brooks (1993) has shown that the susceptibility to pathogens frequently is increased during parasitism.

In summary, this study shows that parasitism of *C.* *flavipes* suppresses cellular immune reactions in encapsulation and nodulation. Although hosts exhibit a recovery of their hemocytic encapsulation capabilities over the course of parasitism by 6 days after oviposition, they never recover reached control levels. The parasitized *D. saccharalis* was highly susceptible to *B. thuringenisis* HD 73 strain when compared with unparasitized *D. saccharalis*.
